# Comparative Efficacy of Finerenone versus Canagliflozin in Patients with Chronic Kidney Disease and Type 2 Diabetes: A Matching-Adjusted Indirect Comparison

**DOI:** 10.3390/jmahp12030014

**Published:** 2024-07-25

**Authors:** David Cherney, Kerstin Folkerts, Paul Mernagh, Mateusz Nikodem, Joerg Pawlitschko, Peter Rossing, Neil Hawkins

**Affiliations:** 1 Department of Medicine, Division of Nephrology, University Health Network, Toronto, ON M5G 2C4, Canada; 2 Bayer AG, 42117 Wuppertal, Germany; 3 Bayer AG, 13353 Berlin, Germany; 4 Putnam, 30-701 Cracow, Poland; 5 ClinStat GmbH, 50354 Huerth, Germany; 6 Steno Diabetes Center Copenhagen, 2730 Copenhagen, Denmark; 7 Department of Clinical Medicine, University of Copenhagen, 2820 Copenhagen, Denmark; 8 School of Health & Wellbeing, University of Glasgow, Glasgow G12 8TB, UK

**Keywords:** chronic kidney disease (CKD), type 2 diabetes (T2D), finerenone, canagliflozin, sodium glucose cotransporter 2 inhibitors (SGLT2i), matching-adjusted indirect comparison (MAIC)

## Abstract

This study aimed to close an evidence gap concerning the relative efficacy of finerenone versus SGLT2is in patients with chronic kidney disease (CKD) and type 2 diabetes (T2D). Canagliflozin was selected as a proxy for the SGLT2i class. Patient-level data of two randomized controlled trials (RCTs) of finerenone (FIDELIO-DKD and FIGARO-DKD) were used alongside aggregated data from CREDENCE, an RCT of canagliflozin. To account for meaningful between-study heterogeneity between each finerenone trial and CREDENCE, a matching-adjusted indirect comparison of a range of efficacy outcomes was undertaken for each finerenone study versus CREDENCE. These results were meta-analyzed, enabling the estimation of the relative effects of finerenone against canagliflozin. For the cardiorenal composite endpoint, the hazard ratio (HR) comparing finerenone to canagliflozin was 1.07 (95% CI: 0.83 to 1.36). The corresponding HRs for all-cause mortality, end-stage kidney disease and cardiovascular death were 0.99 (95% CI: 0.73 to 1.34), 1.03 (95% CI: 0.68 to 1.55) and 0.94 (95% CI: 0.64 to 1.37), respectively. The absence of statistically significant differences was consistent throughout the main analysis and a range of sensitivity analyses. Based on this study, using a large sample of data and adjusted for meaningful differences between the baseline characteristics of the included RCTs, there was no statistically significant evidence indicating a difference in the efficacy of finerenone compared to canagliflozin in the treatment of CKD in patients with T2D.

## 1. Introduction

Treatment goals for patients with chronic kidney disease (CKD) include a delay in disease progression, prevention of end-stage kidney disease (ESKD), and reduction in associated cardiovascular risk [1]. Management of progression and complications of CKD is based on controlling blood pressure and interruption of the renin-angiotensin-aldosterone system [2] and focuses on cardiovascular risk reduction, treatment of albuminuria, and protection of renal functioning [3]. The FIDELIO-DKD (NCT02540993) and FIGARO-DKD (NCT02545049) trials have both demonstrated that finerenone, a nonsteroidal mineralocorticoid receptor antagonist (MRA), reduced the progression of CKD compared to placebo. Finerenone is recommended in guidelines for the management of type 2 diabetes (T2D) with CKD [2,4].

In the course of a pre-specified pooled analysis of FIDELIO-DKD and FIGARO-DKD, sodium-glucose transporter 2 inhibitors (SGLT2i) use grew from 6.7% at baseline to 12.0% [5], reaching as high as 15.8% in FIGARO-DKD [6]. Since then, SGLT2i use can be expected to have grown considerably, with updated guidelines reflecting their important place in the standard of care for CKD in T2D [7]. While the benefits of finerenone on cardiorenal outcomes are known to be independent of concomitant SGLT2i use [8], in the absence of head-to-head RCTs, a data gap remains concerning the relative efficacy of finerenone versus SGLT2is in this indication, particularly in a world in which SGLT2i use exceeds that observed in the finerenone trials. This potentially has implications for both clinicians and payers.

The ability to utilize an indirect treatment comparison (ITC) [9] to address this gap and, therefore, appropriately draw comparisons between the finerenone and SGLT2i trials is compromised by a range of meaningful differences, including variation in inclusion criteria of participants, e.g., estimated glomerular filtration rate (eGFR) and urinary albumin-to-creatinine ratio (UACR) thresholds at screening, differences in baseline population characteristics, concomitant medications used during the trials, and variability in endpoint definitions. Each of these contributes to meaningful between-study heterogeneity, possibly rendering an unadjusted ITC analysis biased. While an earlier study [10] examined the influence of trial design on treatment effects observed in FIDELIO-DKD, demonstrating cardiorenal benefits of the same magnitude between finerenone and canagliflozin, the present study considers a different methodological approach that complements that evidence by also including FIGARO-DKD. Specifically, a matching-adjusted indirect comparison (MAIC) leverages individual patient data from FIDELIO-DKD and FIGARO-DKD, allowing adjustments to be made for differences in baseline populations relative to aggregate-level data from CREDENCE (an SGLT2i trial). By minimizing the impact of heterogeneity on the estimation of treatment effects, we generate a more balanced assessment that emulates a standard ITC based on studies with analogous designs and comparable population characteristics.

By assessing the effect of finerenone relative to the SGLT2is in the treatment of CKD in patients with T2D, this approach fills an important data gap for finerenone.

## 2. Materials and Methods

### 2.1. Study Design

The general approach applied in this study was the following. The baseline characteristics of FIDELIO-DKD and FIGARO-DKD were adjusted, in separate processes of patient-weights assignment, to the respective baseline characteristics of CREDENCE. Then the parameters of relative efficacy of finerenone compared to placebo were estimated for each of weighted FIDELIO-DKD and FIGARO-DKD. Finally, results of a meta-analysis of those two, as well as results of the CREDENCE trial, were used for matching-adjusted indirect comparison between finerenone and canagliflozin.

### 2.2. Source Data

Source data for the MAIC analysis were extracted from three studies. Individual patient data for finerenone were sourced from two clinical studies (FIDELIO-DKD and FIGARO-DKD). Trial-level aggregated data were sourced for canagliflozin from one clinical study (CREDENCE).

Canagliflozin was selected as a proxy for the SGLT2i class. The CREDENCE trial, which reported data for canagliflozin, was the first RCT in this class that considered T2D with CKD. The study population was, therefore, similar to the patient population in both FIDELIO-DKD and FIGARO-DKD, and several endpoints were defined similarly or equivalently, with adjudication of endpoints in the finerenone studies lending credibility. Nevertheless, marked differences remain in the baseline characteristics of each finerenone study versus the CREDENCE population, and this heterogeneity could be adjusted for via the MAIC.

All studies included in this analysis are multicenter, double-blind, placebo-controlled randomized trials. The key inclusion criteria are detailed in Table 1. The FIDELIO-DKD trial predominantly enrolled patients with a high kidney risk, presenting as CKD stage 3 and stage 4 with severely elevated levels of albuminuria, in addition to T2D [11,12], while FIGARO-DKD predominantly included participants with CKD stage 2-4, exhibiting severely elevated albuminuria, alongside T2D and high cardiovascular risk [6,13]. Notably, due to the trial design, individuals with a high cardiovascular risk represented a small proportion of FIDELIO-DKD. Representation of these patients was more substantial in the FIGARO-DKD study. The CKD-related criteria for patient inclusion in CREDENCE differed from those in FIDELIO-DKD and FIGARO-DKD. Specifically, only patients demonstrating very high levels of albuminuria (UACR > 300 mg/g) were eligible for CREDENCE, whereas patients with UACR values ranging between 30 and 300 mg/g were also allowed for inclusion in the two finerenone studies.

### 2.3. Statistical Methods

An MAIC is an approach often considered by health technology assessment agencies [14] to compare treatment effects in the absence of an RCT. As opposed to an unadjusted ITC, an MAIC adjusts for between-trial differences in the distribution of the baseline characteristics that can have an impact on the outcomes [14,15]. In this case, individual patient data from clinical studies on finerenone were compared (separately for FIDELIO-DKD and FIGARO-DKD) with aggregate data from the relevant canagliflozin clinical study (CREDENCE).

A patient-specific weight was then assigned to each participant of the FIDELIO-DKD and FIGARO-DKD studies such that the weighted study-level baseline characteristics for each individual study matched the selected baseline characteristics of the CREDENCE study. Reweighting was based on the estimated propensity of “enrolment” in the finerenone versus canagliflozin trials following a logistic regression model, as described in detail by Signorovitch et al. [15]. Then the distribution of the weights was visually inspected to verify the model fit. This process is similar to propensity score matching [16]; however, here, the weights are not assigned to patients within the CREDENCE trial since only study-level data were available.

The following example provides an illustration of the weight-assignment process for the matching of baseline eGFR. The mean baseline eGFR in FIDELIO-DKD was 44.4 mL/min/1.73 m^2^ versus 56.2 mL/min/1.73 m^2^ in CREDENCE. To adjust the FIDELIO-DKD population to be comparable to the population of CREDENCE, a patient with a baseline eGFR of 40 mL/min/1.73 m^2^ within FIDELIO-DKD, for example, would be assigned a smaller weight than a patient with a baseline eGFR of, say, 55 mL/min/1.73 m^2^ (assuming equality between those two patients regarding all the other matching variables).

The weights assigned to the FIDELIO-DKD and FIGARO-DKD patients were unique (one weight per patient), influencing the survival curves of all of the analyzed endpoints (such that the greater a weight assigned to a participant, the higher the impact on the curve). Consequently, the weights were accounted for in the Cox models used for the estimation of the hazard ratios (HRs) measuring the effect of finerenone over placebo in FIDELIO-DKD and FIGARO-DKD.

The individual study results based on FIDELIO-DKD and FIGARO-DKD were then pooled using a fixed effect meta-analysis (M-A). Finally, the relative effects of finerenone compared to canagliflozin were estimated using the effects of finerenone, as estimated in the previous steps of the MAIC, and the effects of canagliflozin as reported in publications of the CREDENCE study [17]. The analysis was conducted using R Project for Statistical Computing version 4.0.5 (R Foundation for Statistical Computing, Vienna, Austria) and SAS software version 9.4 (SAS Institute).

### 2.4. Outcome Assessment

The outcomes in this study included a range of time-to-event renal and cardiovascular endpoints assessing the time between randomization and the event date. The outcomes analyzed in both the main analysis and the sensitivity analyses included the cardiorenal composite endpoint (kidney failure [defined as end-stage kidney disease or an eGFR of less than 15 mL per minute per 1.73 m^2^, with end-stage kidney disease in turn defined as the initiation of dialysis for ≥90 days or kidney transplantation], doubling of the serum creatinine level and death caused by renal of cardiovascular disease), all-cause mortality, ESKD and cardiovascular death (Table 2). Moreover, six additional endpoints (Table S1) were analyzed following the methodology used in the main analysis.

### 2.5. Analyses

The matching variables of the MAIC model were selected from available baseline characteristics which were, on clinical reasoning, identified as potential treatment effect modifiers. The selection process, ensuring a proper balance between the need to both include all possible effect modifiers and to avoid loss of precision due to over-fitting, was supported by visual inspection of results of subgroup analyses reported for the FIDELIO-DKD and FIGARO-DKD trials [6,11].

In the main analysis, the matching variables were limited to baseline eGFR, UACR, history of CVD and BMI. Five sensitivity analyses, however, examined the impact of an expanded list of matching variables to ensure that all possible effect modifiers were considered (details in Table 3).

The analyses of efficacy endpoints were based on the intention to treat (ITT) population of FIDELIO-DKD and FIGARO-DKD, with two exceptions included in the sensitivity analyses, in which the population from the finerenone studies was restricted to be ‘CREDENCE-eligible’ patients (i.e., including only those patients who, at baseline, were ≥30 years of age, had eGFR ≥ 30 mL/min/1.73 m^2^ and UACR ≥ 300 mg/g, and who were not SGLT2i users).

## 3. Results

### 3.1. Matching of Baseline Characteristics

A total of 5674 patients were analyzed in the FIDELIO-DKD trial [12], and a further 7352 patients were analyzed in FIGARO-DKD [13]. Each of the finerenone studies was matched separately to the CREDENCE trial. For the main analysis, similar populations were obtained following the application of the matching procedure, as evident in the equality of the post-matching variable summaries and the unadjusted values from CREDENCE. Of all variables included in the analysis as matching variables, mean eGFR was the characteristic in FIDELIO-DKD that changed most when comparing post-matching and pre-matching values (Table 4). Moreover, matching also led to marked adjustments in the proportion of patients falling in each of the KDIGO categories by which CKD staging is defined for both eGFR and UACR [18] in FIGARO-DKD (Table 5). The impact of the matching process on other study-level baseline characteristics that were not used as matching variables is also presented for FIDELIO-DKD [11] and FIGARO-DKD [6] in Table S2 and Table S3, respectively.

Of the two finerenone studies, FIGARO-DKD required greater adjustment than FIDELIO-DKD in order to generate a population that matched the population of CREDENCE. This is an expected consequence of the focus of FIGARO-DKD on participants with less advanced CKD and a broader scope of albuminuria (Table 4 and Table 5).

The MAIC analysis also included calculation of the effective sample size (ESS) of each analysis, a measure of precision that considers the impact of applying weights to each patient [15]. As shown in Table 3, after the matching procedure and, in the case of Sensitivity Analysis 3 and Sensitivity Analysis 5, removing those patients who would not be eligible to be enrolled in the CREDENCE trial, the post-matching ESS for all scenarios based on FIDELIO-DKD data ranged from 893 (Sensitivity Analysis 2) to 1288 (main analysis) compared to 5674 patients analyzed in the trial. In the case of the FIGARO-DKD trial, as shown in Table 4, there were 7352 patients analyzed and a range of 170 (Sensitivity Analysis 5) to 1032 (main analysis) for the ESS after matching and removal of ineligible patients in selected sensitivity analyses. While the ESS was substantially smaller compared to the original sample size in both cases, the absolute values remained reasonably high (>500) for the majority of scenarios, including the main analysis. Details on sample sizes and effective sample sizes by scenario are presented in Table S4 for FIDELIO-DKD and Table S5 for FIGARO-DKD.

### 3.2. Outcomes

For the endpoints included in the main analysis, the results of comparisons versus placebo and the results of the MAIC are presented in Table 6 and Table 7, respectively.

For the cardiorenal composite endpoint, the population-adjusted point estimate of the HR finerenone versus placebo was 0.72 with 95% CI (0.59 to 0.90) in the main analysis for FIDELIO-DKD and 0.83 with 95% CI (0.58 to 1.20) for FIGARO-DKD. In the meta-analysis of these trials, the HR was 0.75 with 95% CI (0.62 to 0.90) (Table 6). In CREDENCE, the point estimate of the HR canagliflozin versus placebo for this endpoint was 0.70 with 95% CI (0.59 to 0.82) (Table 6). Accordingly, for the comparison of meta-analyzed finerenone versus canagliflozin, the point estimate of the HR was 1.07 with 95% CI (0.83 to 1.36) (Table 7). At the significance threshold of *p* > 0.05, this result did not represent a statistically significant difference (*p* = 0.61). As shown in Table 7, this was consistent across the endpoints included in the main analysis, with none of the analyzed endpoints reaching a statistically significant difference.

Results of analyses considering additional endpoints are presented in Tables S6 and S7. Supplementary Figures S1–S4 present forest plots for each endpoint included in the analysis, including results based on FIDELIO-DKD, FIGARO-DKD and the meta-analysis of FIDELIO-DKD + FIGARO-DKD. These include results across the main analysis and all variants of the sensitivity analysis. These demonstrate consistency, with all 95% CIs crossing unity except for the ESKD endpoint in FIGARO-DKD in only two sensitivity analyses.

## 4. Discussion

FIDELIO-DKD and FIGARO-DKD compared finerenone with placebo (on top of background therapy in both arms), showing that finerenone is beneficial when used as an add-on to background therapy in patients with CKD and T2D. These benefits were observed in cardiorenal outcomes across studies, irrespective of SGLT2i use [8]. Additionally, since finerenone and SGLT2is rely on different mechanisms of action and are in different drug classes, there is no compelling reason for clinicians to make treatment decisions between finerenone and SGLT2is in the treatment of CKD in patients with T2D. Even so, the relative performance between these treatments may be of interest. While there is an ongoing study assessing the relative impact of UACR [19], there are no RCTs comparing finerenone and SGLT2is on hard endpoints, and such studies are unlikely in the future. Moreover, this means that the use of unadjusted indirect treatment comparisons to draw methodologically valid comparisons between these two classes of drugs on the basis of the published evidence is challenging because the participant characteristics differ between studies.

The present analysis, which applied appropriate adjustment methods, included efficacy endpoints common to the finerenone trials and CREDENCE for which outcomes were available or able to be calculated using patient-level data from FIDELIO-DKD and FIGARO-DKD. These endpoints, four of which were considered in the main analysis and a further six in supplementary analyses, included renal, cardiovascular, and other time-to-event endpoints. There was no evidence of a statistically significant difference between finerenone and canagliflozin for any of these outcomes in patients with CKD and T2D when treatment effect-modifying participant characteristics were balanced and when endpoint definitions were aligned. Moreover, this conclusion was consistent across the range of analyses examining the impact of alternative assumptions regarding treatment effect modifiers and eligibility criteria, with no significant differences between finerenone and canagliflozin in the results of any of the scenarios.

Sensitivity Analysis 5 was conducted in a population limited to participants who would also be eligible to enter the CREDENCE study (i.e., the ‘CREDENCE-eligible’ population). The analysis removed those who were using SGLT2is at baseline in both FIDELIO-DKD and FIGARO-DKD (but kept those participants who started SGLT2is during the study period) and matched additionally for the proportion of participants with a history of heart failure at baseline. This analysis can be considered as providing the most balanced assessment of finerenone and canagliflozin since it considers nearly equivalent populations and treatment with a common comparator. The HR of the cardiorenal endpoint comparing finerenone vs. canagliflozin estimated in this analysis was 0.96 with a 95% CI (0.73; 1.26). Similar results were observed for all additional endpoints considered in the sensitivity analyses; the HR was 0.89 (95% CI: 0.64; 1.25) for all-cause mortality, 0.87 (95% CI: 0.55; 1.38) for ESKD and 0.87 (95% CI: 0.57; 1.34) for CV death.

When interpreting the results of this study, some limitations should be considered. Although an MAIC is an established population adjustment methodology used in health technology assessment [14], it is limited to adjustments to measured baseline characteristics. Consequently, a number of potentially meaningful differences between studies remain. In this analysis, these include the role of SGLT2is in the standard of care, maximally tolerated doses of ACEi/ARB therapy, and the definition of chronic dialysis. The analysis was limited to a comparison with canagliflozin on the basis of the CREDENCE study only. Nonetheless, the evidence across canagliflozin, dapagliflozin and empagliflozin points to an internally and externally consistent class effect of SGLT2is on cardiorenal outcomes [20] and the conclusions based on an MAIC vs. canagliflozin in this indication are, therefore also expected to be valid across the range of SGLT2is. Finally, it is worth noting that a consequence of the process of matching is that the participant population analyzed differs from the original FIDELIO-DKD and FIGARO-DKD trial populations used for registration and regulatory approval. It is also important to note that the scope of this study was limited to efficacy endpoints only.

In many cases, safety endpoints and endpoint definitions did not align sufficiently to allow an MAIC to be undertaken. While there was sufficient overlap in the hyperkalemia endpoints between the trials, an adjusted analysis was not deemed informative since the reduced risk of serious hyperkalemia in people with T2D or with CKD associated with SGLT2is [21] has not been observed with finerenone [6,11]. Any adjustment for differences in baseline characteristics would, therefore, not be expected to impact the relative risk of hyperkalemia between finerenone and canagliflozin. Further, a previous MAIC conducted for FIDELIO-DKD versus CREDENCE [22] showed that adjustment for differences in baseline characteristics did not impact the hyperkalemia HR observed in FIDELIO-DKD. Expansion of the current study to include hyperkalemia was, therefore, not warranted.

In conclusion, based on our analysis using a large sample data and a method that adjusted for meaningful differences between the baseline characteristics of the included studies, no evidence of a difference in the efficacy of finerenone compared to canagliflozin in the treatment of CKD in patients with T2D was observed.

## Figures and Tables

**Table 1:** Key inclusion criteria for studies included in MAIC.

Inclusion Criterion	FIDELIO-DKD	FIGARO-DKD	CREDENCE
Age (years)	18+	18+	30+
Indication	CKD and T2D	CKD and T2D	CKD and T2D
CKD specification	(1) eGFR of 25 to <60 mL/min/1.73 m^2^ and UACR of 30 to 300 mg/g and presence of diabetic retinopathy in the medical history or (2) eGFR of 25 to <75 mL/min/1.73 m^2^ and UACR of 300 to <5000 mg/g	(1) eGFR of 25 to <90 mL/min/1.73 m^2^ and UACR of 30 to 300 mg/g or (2) eGFR of ≥60 mL/min/1.73 m^2^ and UACR of 300 to <5000 mg/g	eGFR of 30 to <90 mL/min/1.73 m^2^ and UACR of 300 to 5000 mg/g
An ACEi or an ARB pretreatment at the maximum tolerated dose	yes	yes	yes (but dual-agent treatment with an ACEi and ARB, direct renin inhibitors, or an MRA is not allowed)
Other	a serum potassium level of 4.8 mmol/L	a serum potassium level of 4.8 mmol/L	

**Table 2:** Endpoints analyzed in the main analysis.

	Endpoint Definition	Type of Endpoint	Estimated Parameter
Cardiorenal composite endpoint	Composite of a, b, c, d: Kidney failure, defined as - dialysis for at least 30 days *, or - kidney transplantation, or - an eGFR of <15 mL/min/1.73 m^2^ sustained for at least 30 days, (of note: in CREDENCE the ‘kidney failure’ component defined above was called ‘ESKD’) b. Doubling of the serum creatinine level from baseline ** sustained for at least 30 days according to central laboratory assessment (equivalent to 57% decline of eGFR, compared to baseline), c. Death from renal disease, or d. Death from CVD.	Time-to-event	HR (95% CI)
All-cause mortality	Death from any reason	Time-to-event	HR (95% CI)
ESKD	Composite endpoint of: - Initiation of long-term dialysis (at least 30 days *) or - Kidney transplantation.	Time-to-event	HR (95% CI)
CV death	CV death, i.e., resulting from an acute MI, sudden cardiac death, death due to heart failure, death due to stroke, death due to CV procedures, death due to CV hemorrhage, and death due to other CV causes	Time-to-event	HR (95% CI)

CI, confidence interval; CV, cardiovascular; CVD, cardiovascular disease; eGFR, estimated glomerular filtration rate; ESKD, end-stage kidney disease; HR, hazard ratio; MI, myocardial infarction. * 90 days in the endpoint definition applied to FIDELIO-DKD and FIGARO-DKD. ** In CREDENCE, the baseline was calculated as the average of randomization and pre-randomization value, while in FIDELIO-DKD and FIGARO-DKD, it was just the pre-randomization value.

**Table 3:** Matching variables included in each analysis.

			Analysis					
Variable Used for Matching	Type of Variable	Matching Statistics	Main	SA1	SA2	SA3	SA4	SA5
eGFR	Continuous	Mean, SD	✓	✓	✓	✓	✓	✓
UACR	Categorical	Proportion of each category	✓	✓	✓	✓	✓	✓
History of CVD	Binary	Proportion of ‘yes’	✓	✓	✓	✓	✓	✓
BMI	Continuous	Mean, SD	✓	✓	✓	✓	✓	✓
Sex	Binary	Proportion of ‘yes’		✓	✓			
Race	Categorical	Proportion of each category		✓	✓			
Age	Continuous	Mean, SD		✓	✓			
Region	Categorical	Proportion of each category			✓			
HbA1c	Continuous	Mean, SD			✓			
Systolic blood pressure	Continuous	Mean, SD			✓			
Use of Sulfonylurea	Binary	Proportion of ‘yes’			✓			
Use of GLP-1 RA	Binary	Proportion of ‘yes’			✓			
Use of diuretics	Binary	Proportion of ‘yes’			✓			
History of HF	Binary	Proportion of ‘yes’					✓	✓
**Population**								
ITT			✓	✓	✓		✓	
Restricted to ‘CREDENCE-eligible’						✓		✓

BMI, body mass index; CVD, cardiovascular disease; eGFR, estimated glomerular filtration rate; GLP-1 RA, glucagon-like peptide-1 receptor agonist; HbA1c, glycosylated hemoglobin type A1c; HF, heart failure; ITT, intention to treat; SA, sensitivity analysis; SD, standard deviation, UACR, urinary albumin-to-creatinine ratio.

**Table 4:** Pre- and post-matching of baseline participant characteristics of FIDELIO-DKD vs. CREDENCE. Variables used in the main analysis.

Variables Included in MAIC	Category/Statistic	FIDELIO-DKD: Pre-Matching			FIDELIO-DKD: Post-Matching			CREDENCE Total
		Finerenone	Placebo	Total	Finerenone	Placebo	Total	
Participants in population	N	2833	2841	5674	NA	NA	NA	4401
ESS	N	NA	NA	NA	643.0	644.9	1287.9	NA
Baseline eGFR (mL/min/1.73 m^2^)	N	2833	2841	5674	643.0	644.9	1287.9	4401
	Mean	44.4	44.3	44.3	55.8	56.6	56.2	56.2
	SD	12.5	12.6	12.6	17.9	18.5	18.2	18.2
Baseline eGFR–distribution (mL/min/1.73 m^2^) ^	<30	342 (12.1%)	354 (12.5%)	696 (12.3%)	31.8 (4.9%)	32.2 (5.0%)	64.0 (5.0%)	174 (4.0%)
	≥30 to <45	1201 (42.4%)	1221 (43.0%)	2422 (42.7%)	152.1 (23.7%)	153.3 (23.8%)	305.5 (23.7%)	1191 (27.1%)
	≥45 to <60	972 (34.3%)	928 (32.7%)	1900 (33.5%)	228.0 (35.5%)	211.1 (32.7%)	438.9 (34.1%)	1266 (28.8%)
	≥60 to <90	313 (11.0%)	332 (11.7%)	645 (11.4%)	198.9 (30.9%)	202.5 (31.4%)	401.5 (31.2%)	1558 (35.4%)
	≥90	5 (0.2%)	6 (0.2%)	11 (0.2%)	32.2 (5.0%)	45.7 (7.1%)	78.1 (6.1%)	211 (4.8%)
Baseline UACR (macroalbuminuria status; mg/g)	≤300	361 (12.7%)	347 (12.2%)	708 (12.5%)	77.3 (12.0%)	77.3 (12.0%)	154.5 (12.0%)	527 (12.0%)
	>300 to ≤3000	2267 (80.0%)	2275 (80.1%)	4542 (80.0%)	498.1 (77.5%)	488.6 (75.8%)	986.5 (76.6%)	3371 (76.6%)
	>3000	205 (7.2%)	219 (7.7%)	424 (7.5%)	67.7 (10.5%)	79.0 (12.2%)	146.8 (11.4%)	503 (11.4%)
History of CVD at baseline	Yes	1303 (46.0%)	1302 (45.8%)	2605 (45.9%)	312.3 (48.6%)	336.5 (52.2%)	649.1 (50.4%)	2220 (50.4%)
	No	1530 (54.0%)	1539 (54.2%)	3069 (54.1%)	330.7 (51.4%)	308.3 (47.8%)	638.8 (49.6%)	2181 (49.6%)
Baseline BMI (kg/m^2^)	N	2833	2841	5674	643.0	644.9	1287.9	4401
	Mean	31.1	31.1	31.1	31.3	31.3	31.3	31.3
	SD	6.0	6.0	6.0	6.0	6.4	6.2	6.2

BMI, body mass index; CVD, cardiovascular disease; eGFR, estimated glomerular filtration rate; ESS, effective sample size; MAIC, matching-adjusted indirect comparison; N, number; NA, not applicable; SD, standard deviation; UACR, urinary albumin-to-creatinine ratio. ^ not considered as a matching variable, i.e., the matching process did not aim to equalize the proportion of FIDELIO-DKD patients in each category with CREDENCE.

**Table 5:** Pre- and post-matching of baseline participant characteristics of FIGARO-DKD vs. CREDENCE. Variables used in the main analysis.

Variables Included in the MAIC	Category/Statistic	FIGARO-DKD: Pre-Matching			FIGARO-DKD: Post-Matching			CREDENCE Total
		Finerenone	Placebo	Total	Finerenone	Placebo	Total	
Participants in population	N	3686	3666	7352	NA	NA	NA	4401
ESS	N	NA	NA	NA	517.6	514.8	1032.4	NA
Baseline eGFR (mL/min/1.73 m^2^)	N	3686	3666	7352	517.6	514.8	1032.4	4401
	Mean	67.6	68.0	67.8	56.7	55.8	56.2	56.2
	SD	21.6	21.7	21.7	18.2	18.2	18.2	18.2
Baseline eGFR–distribution (mL/min/1.73 m^2^) ^	<30	98 (2.7%)	96 (2.6%)	194 (2.6%)	40.8 (7.9%)	45.0 (8.7%)	85.9 (8.3%)	174 (4.0%)
	≥30 to <45	558 (15.1%)	526 (14.3%)	1084 (14.7%)	106.9 (20.7%)	124.0 (24.1%)	231.4 (22.4%)	1191 (27.1%)
	≥45 to <60	745 (20.2%)	789 (21.5%)	1534 (20.9%)	133.6 (25.8%)	115.0 (22.3%)	248.2 (24.0%)	1266 (28.8%)
	≥60 to <90	1631 (44.2%)	1601 (43.7%)	3232 (44.0%)	218.0 (42.1%)	214.4 (41.6%)	432.3 (41.9%)	1558 (35.4%)
	≥90	654 (17.7%)	654 (17.8%)	1308 (17.8%)	18.3 (3.5%)	16.3 (3.2%)	34.6 (3.4%)	211 (4.8%)
Baseline UACR (macroalbuminuria status; mg/g)	≤300	1836 (49.8%)	1788 (48.8%)	3624 (49.3%)	65.3 (12.6%)	58.7 (11.4%)	123.9 (12.0%)	527 (12.0%)
	>300 to ≤3000	1769 (48.0%)	1798 (49.0%)	3567 (48.5%)	396.7 (76.6%)	394.1 (76.6%)	790.8 (76.6%)	3371 (76.6%)
	>3000	81 (2.2%)	80 (2.2%)	161 (2.2%)	55.5 (10.7%)	62.0 (12.0%)	117.7 (11.4%)	503 (11.4%)
History of CVD at baseline	Yes	1676 (45.5%)	1654 (45.1%)	3330 (45.3%)	257.3 (49.7%)	262.8 (51.1%)	520.3 (50.4%)	2220 (50.4%)
	No	2010 (54.5%)	2012 (54.9%)	4022 (54.7%)	260.3 (50.3%)	252.0 (48.9%)	512.1 (49.6%)	2181 (49.6%)
Baseline BMI (kg/m^2^)	N	3686	3666	7352	517.6	514.8	1032.4	4401
	Mean	31.5	31.4	31.4	31.4	31.2	31.3	31.3
	SD	6.0	5.9	6.0	6.5	5.9	6.2	6.2

BMI, body mass index; CVD, cardiovascular disease; eGFR, estimated glomerular filtration rate; ESS, effective sample size; MAIC, matching-adjusted indirect comparison; N, number; NA, not applicable; SD, standard deviation; UACR, urinary albumin-to-creatinine ratio. ^ not considered as a matching variable, i.e., the matching process did not aim to equalize the proportion of FIDELIO-DKD patients in each category with CREDENCE.

**Table 6:** Finerenone and canagliflozin vs. placebo: results of weighted analysis.

Endpoint	Finerenone vs. Placebo		Canagliflozin vs. Placebo	
	HR (95% CI) MAIC-Weighted FIDELIO-DKD	HR (95% CI) MAIC-Weighted FIGARO-DKD	HR (95% CI) M-A of FIDELIO-DKD and FIGARO-DKD	HR (95% CI), CREDENCE
Cardiorenal composite endpoint	0.72 (0.59; 0.90)	0.83 (0.58; 1.20)	0.75 (0.62; 0.90)	0.70 (0.59; 0.82)
All-cause mortality	0.84 (0.64; 1.10)	0.79 (0.54; 1.15)	0.82 (0.66; 1.03)	0.83 (0.68; 1.02)
End-stage kidney disease	0.86 (0.64; 1.16)	0.34 (0.16; 0.74)	0.76 (0.58; 1.00)	0.74 (0.55; 1.00)
CV death	0.75 (0.53; 1.07)	0.70 (0.42; 1.15)	0.73 (0.55; 0.98)	0.78 (0.61; 1.00)

CI, confidence interval; CV, cardiovascular; HR, hazard ratio; M-A, meta-analysis; MAIC, matching-adjusted indirect comparison. Endpoint definitions as in Table 2. The HRs estimated for FIDELIO-DKD and FIGARO-DKD were based on a Cox proportional hazards model using the same covariates as in the respective finerenone study but applying a robust (“sandwich”) method of covariance estimation.

**Table 7:** Finerenone (FIDELIO-DKD + FIGARO-DKD) and canagliflozin results of MAIC.

Endpoint	MAIC: Finerenone vs. Canagliflozin	
	HR (95% CI)	*p*-Value
Cardiorenal composite endpoint	1.07 (0.83; 1.36)	0.610
All-cause mortality	0.99 (0.73; 1.34)	0.954
End-stage kidney disease	1.03 (0.68; 1.55)	0.890
CV death	0.94 (0.64; 1.37)	0.750

CV, cardiovascular; CI, confidence interval; HR, hazard ratio; MAIC, matching-adjusted indirect comparison. Endpoint definitions as in Table 2.

## Data Availability

As such, Bayer AG commits to sharing upon request from qualified scientific and medical re-searchers, patient-level clinical trial data, study-level clinical trial data, and protocols from clinical trials in patients for medicines and indications approved in the United States (US) and European Union (EU) as necessary for conducting legitimate research. This applies to data on new medicines and indications that have been approved by the EU and US regulatory agencies on or after 1 January 2014. Interested researchers can use www.vivli.org to request access to anonymized patient-level data and supporting documents from clinical studies to conduct further research that can help advance medical science or improve patient care. Information on the Bayer criteria for listing studies and other relevant information is provided in the member section of the portal. Data access will be granted to anonymized patient-level data, protocols, and clinical study reports after approval by an independent scientific review panel. Bayer is not involved in the decisions made by the independent review panel. Bayer AG will take all necessary measures to ensure that patient privacy is safeguarded.

## Supplementary Materials

The following supporting information can be downloaded at https://www.mdpi.com/article/10.3390/jmahp12030014/s1**,** Table S1: Additional endpoints analyzed (comparison with canagliflozin), Table S2: Pre- and post-matching of baseline participant characteristics of FIDELIO-DKD vs. CREDENCE. Additional variables not used in the main analysis, Table S3: Pre- and post-matching of baseline participant characteristics of FIGARO-DKD vs. CREDENCE. Additional variables not used in the main analysis, Table S4: Sample size and effective sample size of FIDELIO-DKD by analysis, Table S5: Sample size and effective sample size of FIGARO-DKD by analysis. Table S6: Finerenone and canagliflozin vs. placebo: results of weighted analysis, additional endpoints, Table S7: Finerenone (FIDELIO-DKD + FIGARO-DKD) and canagliflozin results of MAIC, additional endpoints, Figure S1: Forest plot of MAIC finerenone vs. canagliflozin for renal/cardiovascular composite endpoint, Figure S2: Forest plot of MAIC finerenone vs. canagliflozin for all-cause mortality endpoint, Figure S3: Forest plot of MAIC finerenone vs. canagliflozin for end-stage kidney disease endpoint, Figure S4: Forest plot of MAIC finerenone vs. canagliflozin for cardiovascular death endpoint.
