# Supplementary material for: Comparative Efficacy of Finerenone versus Canagliflozin in Patients with Chronic Kidney Disease and Type 2 Diabetes: A Matching-Adjusted Indirect Comparison
Source: J Mark Access Health Policy. 2024 Jul 25;12(3):169–80. doi: 10.3390/jmahp12030014 (PMC11348111; doi:10.3390/jmahp12030014)
Supplement: Supplementary file 1 [file jmahp-12-00014-s001.zip › jmahp-3070690-supplementary.pdf]

Journal of Market Access & Health Policy

MDPI

Table S1: Additional endpoints analyzed (comparison with canagliflozin)

Endpoint	Endpoint definition	Type of endpoint	Estimated parameter
– CV composite endpoint	Composite of a,b,c,d,e:<br>a. CV death,<br>b. non-fatal MI (first occurrence of),<br>c. non-fatal stroke (first occurrence of) or<br>d. hospitalization for HF (first occurrence of) or<br>e. unstable angina.	Time-to-event	HR (95% CI)
– Renal composite endpoint	Composite endpoint of a,b,c:<br>a. Kidney failure, defined as:<br>- dialysis for at least 30 days, or<br>- kidney transplantation, or<br>- an eGFR of < 15 mL/min/1.73 m^2 sustained for at least 30 days,<br>b. doubling of the serum creatinine level from baseline (average of randomization and pre-randomization value) sustained for at least 30 days according to the central laboratory assessment (equivalent to 57% decline of eGFR, compared to the baseline), or<br>c. death from renal disease.	Time-to-event	HR (95% CI)
– Sustained eGFR decrease to < 15 mL/min/1.73 m^2	eGFR of < 15 mL/min/1.73 m^2 sustained for at least 30 days	Time-to-event	HR (95% CI)
– Non-fatal stroke	Event of non-fatal stroke	Time-to-event	HR (95% CI)
– HF hospitalization	Event of hospitalization due to HF	Time-to-event	HR (95% CI)
– Non-fatal MI	Event of non-fatal MI	Time-to-event	HR (95% CI)

CI, confidence interval; CV, cardiovascular; eGFR, estimated glomerular filtration rate; HF, heart failure; HR, hazard ratio; MI, myocardial infarction.

Table S2: Pre- and post-matching of baseline participant characteristics of FIDELIO-DKD vs CREDENCE. Additional variables not used in Main Analysis.

Other baseline characteristics, not included in MAIC	Category/Statistic	FIDELIO-DKD - before matching			FIDELIO-DKD - after matching			CREDENCE Total
		Finerenone	Placebo	Total	Finerenone	Placebo	Total	
Participants in population	N	2833	2841	5674	NA	NA	NA	4401
ESS	N	NA	NA	NA	643.0	644.9	1287.9	NA
Sex	Male	1953 (68.9%)	2030 (71.5%)	3983 (70.2%)	439.4 (68.3%)	448.7 (69.6%)	888.1 (69.0%)	2907 (66.1%)
	Female	880 (31.1%)	811 (28.5%)	1691 (29.8%)	203.7 (31.7%)	196.2 (30.4%)	399.8 (31.0%)	1494 (33.9%)
Race	White	1777 (62.7%)	1815 (63.9%)	3592 (63.3%)	425.7 (66.2%)	424.4 (65.8%)	850.1 (66.0%)	2931 (66.6%)
	Asian	717 (25.3%)	723 (25.4%)	1440 (25.4%)	139.5 (21.7%)	140.8 (21.8%)	280.4 (21.8%)	877 (19.9%)
	Black	140 (4.9%)	124 (4.4%)	264 (4.7%)	27.3 (4.2%)	26.2 (4.1%)	53.5 (4.2%)	224 (5.1%)
	Other	199 (7.0%)	179 (6.3%)	378 (6.7%)	50.5 (7.9%)	53.4 (8.3%)	104.0 (8.1%)	369 (8.4%)
Age (years)	N	2833	2841	5674	643.0	644.9	1287.9	4401
	Mean	65.4	65.7	65.6	64.3	64.5	64.4	63.0
	SD	8.9	9.2	9.1	9.3	9.4	9.3	9.2
	Median	66.0	66.0	66.0	65.0	66.0	65.0	NR
	IQR	12.0	12.0	12.0	12.0	12.0	12.0	NR
Region	Europe	1182 (41.7%)	1176 (41.4%)	2358 (41.6%)	303.8 (47.2%)	292.2 (45.3%)	595.9 (46.3%)	864 (19.6%)
	North America	467 (16.5%)	477 (16.8%)	944 (16.6%)	98.8 (15.4%)	103.0 (16.0%)	201.8 (15.7%)	1182 (26.9%)
	Latin America	295 (10.4%)	298 (10.5%)	593 (10.5%)	70.0 (10.9%)	78.6 (12.2%)	148.6 (11.5%)	941 (21.4%)

Other baseline characteristics, not included in MAIC	Category/Statistic	FIDELIO-DKD - before matching			FIDELIO-DKD - after matching			CREDENCE Total
		Finerenone	Placebo	Total	Finerenone	Placebo	Total	
	Other	889 (31.4%)	890 (31.3%)	1779 (31.4%)	170.5 (26.5%)	171.1 (26.5%)	341.6 (26.5%)	1414 (32.1%)
Baseline HbA1c (%)	N	2833	2841	5674	643.0	644.9	1287.9	4401
	Mean	7.7	7.7	7.7	7.8	7.7	7.7	8.3
	SD	1.3	1.4	1.3	1.4	1.4	1.4	1.3
	Median	7.5	7.5	7.5	7.6	7.5	7.5	NR
	IQR	1.8	1.8	1.8	1.8	1.9	1.9	NR
Baseline systolic blood pressure (mmHg)	N	2833	2841	5674	643.0	644.9	1287.9	4401
	Mean	138.0	138.0	138.0	138.0	138.6	138.3	140
	SD	14.3	14.4	14.4	13.6	13.9	13.7	15.6
	Median	138.3	138.3	138.3	137.7	138.7	138.3	NR
	IQR	19.0	19.7	19.3	17.3	18.3	18.3	NR
Use of sulfonylurea at baseline	Yes	654 (23.1%)	673 (23.7%)	1327 (23.4%)	148.9 (23.2%)	156.4 (24.3%)	305.4 (23.7%)	1268 (28.8%)
	No	2179 (76.9%)	2168 (76.3%)	4347 (76.6%)	494.1 (76.8%)	488.5 (75.7%)	982.5 (76.3%)	3133(71.2%)
Use of GLP-1 RA at baseline	Yes	189 (6.7%)	205 (7.2%)	394 (6.9%)	37.7 (5.9%)	45.7 (7.1%)	83.5 (6.5%)	183 (4.2%)
	No	2644 (93.3%)	2636 (92.8%)	5280 (93.1%)	605.4 (94.1%)	599.1 (92.9%)	1204.4 (93.5%)	4218 (95.8%)
Use of diuretics at baseline	Yes	1577 (55.7%)	1637 (57.6%)	3214 (56.6%)	335.2 (52.1%)	331.6 (51.4%)	666.7 (51.8%)	2057 (46.7%)
	No	1256 (44.3%)	1204 (42.4%)	2460 (43.4%)	307.9 (47.9%)	313.3 (48.6%)	621.2 (48.2%)	2344 (53.3%)
History of HF at baseline	Yes	195 (6.9%)	241 (8.5%)	436 (7.7%)	57.2 (8.9%)	51.1 (7.9%)	108.3 (8.4%)	652 (14.8 %)
	No	2638 (93.1%)	2600 (91.5%)	5238 (92.3%)	585.8 (91.1%)	593.8 (92.1%)	1179.6 (91.6%)	3749 (85.2%)

Other baseline characteristics, not included in MAIC	Category/Statistic	FIDELIO-DKD - before matching			FIDELIO-DKD - after matching			CREDENCE Total
		Finerenone	Placebo	Total	Finerenone	Placebo	Total	
eGFR - distribution	< 30	342 (12.1%)	354 (12.5%)	696 (12.3%)	31.8 (4.9%)	32.2 (5.0%)	64.0 (5.0%)	174 (4.0%)
	≥ 30 to < 45	1201 (42.4%)	1221 (43.0%)	2422 (42.7%)	152.1 (23.7%)	153.3 (23.8%)	305.5 (23.7%)	1191 (27.1%)
	≥ 45 to < 60	972 (34.3%)	928 (32.7%)	1900 (33.5%)	228.0 (35.5%)	211.1 (32.7%)	438.9 (34.1%)	1266 (28.8%)
	≥ 60 to < 90	313 (11.0%)	332 (11.7%)	645 (11.4%)	198.9 (30.9%)	202.5 (31.4%)	401.5 (31.2%)	1558 (35.4%)
	≥ 90	5 (0.2%)	6 (0.2%)	11 (0.2%)	32.2 (5.0%)	45.7 (7.1%)	78.1 (6.1%)	211 (4.8%)
Duration of diabetes (years)	N	2833	2841	5674	643.0	644.9	1287.9	4401
	Mean	16.6	16.5	16.6	16.0	16.1	16.0	15.8
	SD	8.8	8.8	8.8	8.6	8.6	8.6	8.6
	Median	16.1	16.2	16.1	15.1	15.3	15.2	NR
	IQR	11.1	11.1	11.1	11.0	11.3	11.0	NR
UACR (median, quartiles; mg/g)	N	2833	2841	5674	643.0	644.9	1287.9	4401
	Mean	1185.9	1210.3	1198.1	1252.6	1283.5	1268.3	NR
	SD	1036.5	1052.2	1044.4	1123.4	1182.5	1153.8	NR
	Median	833.1	868.1	852.5	860.4	864.5	862.5	927
	Q1-Q3	441.5 - 1625.0	453.8 - 1642.2	446.3 - 1633.8	466.0 - 1651.6	440.2 - 1673.4	450.0 - 1657.8	463–1833
Serum potassium — mmol/litter	N	2833	2841	5674	643.0	644.9	1287.9	4401
	Mean	4.37	4.38	4.37	4.35	4.34	4.34	NR
	SD	0.45	0.46	0.46	0.46	0.46	0.46	NR
	Median	4.40	4.40	4.40	4.40	4.30	4.30	NR
	IQR	0.60	0.60	0.60	0.50	0.60	0.50	NR
Use of ACEi at baseline	Yes	950 (33.5%)	992 (34.9%)	1942 (34.2%)	230.8 (35.9%)	230.9 (35.8%)	461.7 (35.8%)	NR

Other baseline characteristics, not included in MAIC	Category/Statistic	FIDELIO-DKD - before matching			FIDELIO-DKD - after matching			CREDENCE Total
		Finerenone	Placebo	Total	Finerenone	Placebo	Total	
	No	1883 (66.5%)	1849 (65.1%)	3732 (65.8%)	412.3 (64.1%)	414.0 (64.2%)	826.2 (64.2%)	NR
Use of ARB at baseline	Yes	1879 (66.3%)	1846 (65.0%)	3725 (65.7%)	410.4 (63.8%)	412.5 (64.0%)	822.9 (63.9%)	NR
	No	954 (33.7%)	995 (35.0%)	1949 (34.3%)	232.6 (36.2%)	232.4 (36.0%)	465.0 (36.1%)	NR
Use of statin at baseline	Yes	2105 (74.3%)	2110 (74.3%)	4215 (74.3%)	465.0 (72.3%)	461.1 (71.5%)	926.1 (71.9%)	3036 (69.0%)
	No	728 (25.7%)	731 (25.7%)	1459 (25.7%)	178.0 (27.7%)	183.7 (28.5%)	361.8 (28.1%)	1365 (31.0%)
Use of potassium-lowering agent at baseline	Yes	70 (2.5%)	66 (2.3%)	136 (2.4%)	13.1 (2.0%)	10.1 (1.6%)	23.1 (1.8%)	NR
	No	2763 (97.5%)	2775 (97.7%)	5538 (97.6%)	630.0 (98.0%)	634.8 (98.4%)	1264.8 (98.2%)	NR
Use of any glucose-lowering therapy at baseline	Yes	2747 (97.0%)	2777 (97.7%)	5524 (97.4%)	626.8 (97.5%)	635.3 (98.5%)	1262.2 (98.0%)	NR
	No	86 (3.0%)	64 (2.3%)	150 (2.6%)	16.2 (2.5%)	9.5 (1.5%)	25.7 (2.0%)	NR
Use of insulin at baseline	Yes	1843 (65.1%)	1794 (63.1%)	3637 (64.1%)	421.7 (65.6%)	391.2 (60.7%)	812.6 (63.1%)	2884 (65.5%)
	No	990 (34.9%)	1047 (36.9%)	2037 (35.9%)	221.3 (34.4%)	253.7 (39.3%)	475.3 (36.9%)	1517(34.5%)
Use of SGLT-2i at baseline	Yes	124 (4.4%)	135 (4.8%)	259 (4.6%)	34.7 (5.4%)	41.1 (6.4%)	75.9 (5.9%)	0
	No	2709 (95.6%)	2706 (95.2%)	5415 (95.4%)	608.3 (94.6%)	603.8 (93.6%)	1212.0 (94.1%)	4401 (100%)

ACEi, angiotensin converting enzyme inhibitor; ARB, angiotensin-receptor blocker; eGFR, estimated glomerular filtration rate; ESS, effective sample size; GLP-1 RA, glucagon-like peptide-1 receptor agonist; HbA1c, glycosylated hemoglobin; HF, heart failure; IQR, interquartile range; MAIC, matching adjusted indirect comparison; NA, not applicable; NR, not reported; Q1-Q3, first-third quartile; SBP, systolic blood pressure; SD, standard deviation; SGLT-2i, sodium-glucose cotransporter-2 inhibitor; UACR, urinary albumin-to-creatinine ratio

Table S3 : Pre- and post-matching of baseline participant characteristics of FIGARO-DKD vs CREDENCE. Additional variables not used in the Main Analysis.

**Other baseline characteristics, not included in the MAIC**	**Category/Statistic**	**FIGARO-DKD - before matching**			**FIGARO-DKD - after matching**			**CREDENCE Total**
		**Finerenone**	**Placebo**	**Total**	**Finerenone**	**Placebo**	**Total**	
Participants in population	N	3686	3666	7352	NA	NA	NA	4401
ESS	N	NA	NA	NA	517.6	514.8	1032.4	NA
Sex	Male	2528 (68.6%)	2577 (70.3%)	5105 (69.4%)	367.9 (71.1%)	351.6 (68.3%)	719.1 (69.7%)	2907 (66.1%)
	Female	1158 (31.4%)	1089 (29.7%)	2247 (30.6%)	149.7 (28.9%)	163.2 (31.7%)	313.3 (30.3%)	1494 (33.9%)
Race	White	2672 (72.5%)	2605 (71.1%)	5277 (71.8%)	356.3 (68.8%)	330.2 (64.1%)	685.8 (66.4%)	2931 (66.6%)
	Asian	715 (19.4%)	739 (20.2%)	1454 (19.8%)	103.0 (19.9%)	117.8 (22.9%)	221.2 (21.4%)	877 (19.9%)
	Black	113 (3.1%)	145 (4.0%)	258 (3.5%)	17.3 (3.4%)	34.7 (6.7%)	52.5 (5.1%)	224 (5.1%)
	Other	186 (5.0%)	177 (4.8%)	363 (4.9%)	41.0 (7.9%)	32.1 (6.2%)	72.9 (7.1%)	369 (8.4%)
Age (years)	N	3686	3666	7352	517.6	514.8	1032.4	4401
	Mean	64.1	64.1	64.1	64.6	64.4	64.5	63.0
	SD	9.7	10.0	9.8	9.3	10.0	9.6	9.2
	Median	65.0	65.0	65.0	66.0	65.0	66.0	NR
	IQR	13.0	13.0	13.0	12.0	14.0	13.0	NR
Region	Europe	1754 (47.6%)	1750 (47.7%)	3504 (47.7%)	243.9 (47.1%)	217.7 (42.3%)	460.8 (44.6%)	864 (19.6%)
	North America	559 (15.2%)	548 (14.9%)	1107 (15.1%)	76.1 (14.7%)	91.0 (17.7%)	167.4 (16.2%)	1182 (26.9%)
	Latin America	424 (11.5%)	417 (11.4%)	841 (11.4%)	67.4 (13.0%)	66.1 (12.8%)	133.5 (12.9%)	941 (21.4%)
	Other	949 (25.7%)	951 (25.9%)	1900 (25.8%)	130.3 (25.2%)	140.1 (27.2%)	270.6 (26.2%)	1414 (32.1%)

Other baseline characteristics, not included in the MAIC	Category/Statistic	FIGARO-DKD - before matching			FIGARO-DKD - after matching			CREDENCE Total
		Finerenone	Placebo	Total	Finerenone	Placebo	Total	
Baseline HbA1c (%)	N	3686	3666	7352	517.6	514.8	1032.4	4401
	Mean	7.7	7.7	7.7	7.7	7.7	7.7	8.3
	SD	1.4	1.3	1.4	1.4	1.4	1.4	1.3
	Median	7.5	7.5	7.5	7.5	7.5	7.5	NR
	IQR	1.9	1.8	1.8	1.9	1.8	1.9	NR
Baseline systolic blood pressure (mmHg)	N	3686	3666	7352	517.6	514.8	1032.4	4401
	Mean	135.8	135.7	135.8	136.8	136.2	136.5	140
	SD	14.0	14.1	14.0	13.6	14.0	13.8	15.6
	Median	135.7	136.0	136.0	136.3	136.7	136.7	NR
	IQR	18.7	19.3	18.7	18.3	19.0	18.7	NR
Use of sulfonylurea at baseline	Yes	1037 (28.1%)	1025 (28.0%)	2062 (28.0%)	130.3 (25.2%)	133.0 (25.8%)	263.4 (25.5%)	1268 (28.8%)
	No	2649 (71.9%)	2641 (72.0%)	5290 (72.0%)	387.3 (74.8%)	381.8 (74.2%)	769.0 (74.5%)	3133(71.2%)
Use of GLP-1 RA at baseline	Yes	308 (8.4%)	242 (6.6%)	550 (7.5%)	35.4 (6.8%)	31.3 (6.1%)	66.6 (6.5%)	183 (4.2%)
	No	3378 (91.6%)	3424 (93.4%)	6802 (92.5%)	482.2 (93.2%)	483.5 (93.9%)	965.8 (93.5%)	4218 (95.8%)
Use of diuretics at baseline	Yes	1748 (47.4%)	1748 (47.7%)	3496 (47.6%)	249.7 (48.2%)	273.0 (53.0%)	523.4 (50.7%)	2057 (46.7%)
	No	1938 (52.6%)	1918 (52.3%)	3856 (52.4%)	267.9 (51.8%)	241.8 (47.0%)	509.0 (49.3%)	2344 (53.3%)
History of HF at baseline	Yes	290 (7.9%)	281 (7.7%)	571 (7.8%)	44.4 (8.6%)	50.1 (9.7%)	94.7 (9.2%)	652 (14.8 %)
	No	3396 (92.1%)	3385 (92.3%)	6781 (92.2%)	473.2 (91.4%)	464.7 (90.3%)	937.7 (90.8%)	3749 (85.2%)
eGFR – distribution (mL/min/1.73 m²)	< 30	98 (2.7%)	96 (2.6%)	194 (2.6%)	40.8 (7.9%)	45.0 (8.7%)	85.9 (8.3%)	174 (4.0%)
	≥ 30 to < 45	558 (15.1%)	526 (14.3%)	1084 (14.7%)	106.9 (20.7%)	124.0 (24.1%)	231.4 (22.4%)	1191 (27.1%)
	≥ 45 to < 60	745 (20.2%)	789 (21.5%)	1534 (20.9%)	133.6 (25.8%)	115.0 (22.3%)	248.2 (24.0%)	1266 (28.8%)
	≥ 60 to < 90	1631 (44.2%)	1601 (43.7%)	3232 (44.0%)	218.0 (42.1%)	214.4 (41.6%)	432.3 (41.9%)	1558 (35.4%)

Other baseline characteristics, not included in the MAIC	Category/Statistic	FIGARO-DKD - before matching			FIGARO-DKD - after matching			CREDENCE Total
		Finerenone	Placebo	Total	Finerenone	Placebo	Total	
	≥ 90	654 (17.7%)	654 (17.8%)	1308 (17.8%)	18.3 (3.5%)	16.3 (3.2%)	34.6 (3.4%)	211 (4.8%)
Duration of diabetes (years)	N	3686	3666	7352	517.6	514.8	1032.4	4401
	Mean	14.5	14.4	14.5	14.6	15.2	14.9	15.8
	SD	8.6	8.4	8.5	8.2	8.7	8.5	8.6
	Median	13.2	13.9	13.3	13.7	14.5	14.1	NR
	IQR	12.0	11.1	11.6	11.1	11.1	11.6	NR
UACR (median, quartiles; mg/g)	N	3686	3666	7352	517.6	514.8	1032.4	4401
	Mean	586.4	586.9	586.7	1076.2	1031.9	1053.5	NR
	SD	764.6	752.4	758.5	1150.7	1115.9	1133.2	NR
	Median	302.4	315.1	308.2	611.8	514.2	561.0	927
	Q1-Q3	105.5 - 749.0	111.3 - 731.0	108.1 - 739.3	354.1 - 1325.1	341.6 - 1272.9	345.9 - 1299.4	463–1833
Serum potassium — mmol/litter	N	3686	3666	7352	517.6	514.8	1032.4	4401
	Mean	4.33	4.33	4.33	4.35	4.38	4.37	NR
	SD	0.43	0.43	0.43	0.45	0.46	0.46	NR
	Median	4.30	4.30	4.30	4.40	4.40	4.40	NR
	IQR	0.50	0.50	0.50	0.60	0.60	0.60	NR
Use of ACEi at baseline	Yes	1576 (42.8%)	1561 (42.6%)	3137 (42.7%)	217.0 (41.9%)	202.3 (39.3%)	418.9 (40.6%)	NR
	No	2110 (57.2%)	2105 (57.4%)	4215 (57.3%)	300.6 (58.1%)	312.5 (60.7%)	613.5 (59.4%)	NR
Use of ARB at baseline	Yes	2108 (57.2%)	2104 (57.4%)	4212 (57.3%)	300.3 (58.0%)	312.3 (60.7%)	613.0 (59.4%)	NR
	No	1578 (42.8%)	1562 (42.6%)	3140 (42.7%)	217.3 (42.0%)	202.5 (39.3%)	419.4 (40.6%)	NR
Use of statin at baseline	Yes	2552 (69.2%)	2632 (71.8%)	5184 (70.5%)	360.5 (69.6%)	383.1 (74.4%)	744.3 (72.1%)	3036 (69.0%)
	No	1134 (30.8%)	1034 (28.2%)	2168 (29.5%)	157.1 (30.4%)	131.7 (25.6%)	288.1 (27.9%)	1365 (31.0%)
Use of potassium-lowering agent at baseline	Yes	24 (0.7%)	22 (0.6%)	46 (0.6%)	1.8 (0.3%)	2.7 (0.5%)	4.5 (0.4%)	NR
	No	3662 (99.3%)	3644 (99.4%)	7306 (99.4%)	515.8 (99.7%)	512.1 (99.5%)	1027.9 (99.6%)	NR

Other baseline characteristics, not included in the MAIC	Category/Statistic	FIGARO-DKD - before matching			FIGARO-DKD - after matching			CREDENCE Total
		Finerenone	Placebo	Total	Finerenone	Placebo	Total	
Use of any glucose-lowering therapy at baseline	Yes	3607 (97.9%)	3589 (97.9%)	7196 (97.9%)	503.8 (97.3%)	495.7 (96.3%)	999.3 (96.8%)	NR
	No	79 (2.1%)	77 (2.1%)	156 (2.1%)	13.8 (2.7%)	19.1 (3.7%)	33.1 (3.2%)	NR
Use of insulin at baseline	Yes	2023 (54.9%)	1970 (53.7%)	3993 (54.3%)	318.7 (61.6%)	293.1 (56.9%)	611.1 (59.2%)	2884 (65.5%)
	No	1663 (45.1%)	1696 (46.3%)	3359 (45.7%)	198.9 (38.4%)	221.7 (43.1%)	421.3 (40.8%)	1517 (34.5%)
Use of SGLT-2i at baseline	Yes	314 (8.5%)	304 (8.3%)	618 (8.4%)	35.5 (6.9%)	33.9 (6.6%)	69.4 (6.7%)	0
	No	3372 (91.5%)	3362 (91.7%)	6734 (91.6%)	482.1 (93.1%)	480.9 (93.4%)	963.0 (93.3%)	4401 (100%)

ACEi, angiotensin converting enzyme inhibitor; ARB, angiotensin-receptor blocker; eGFR, estimated glomerular filtration rate; ESS, effective sample size; GLP-1 RA, glucagon-like peptide-1 receptor agonist; HbA1c, glycosylated hemoglobin; HF, heart failure; IQR, interquartile range; MAIC, matching-adjusted indirect comparison; NA, not applicable; NR, not reported; Q1-Q3, first-third quartile; SBP, systolic blood pressure; SD, standard deviation; SGLT-2i, sodium-glucose cotransporter-2 inhibitor; UACR, urinary albumin-to-creatinine ratio

Table S4: Sample size and effective sample size of FIDELIO-DKD by analysis

	Sample size of the included FIDELIO-DKD population	Effective sample size	Effective sample size /population
Main Analysis	5,674	1,287.9	22.7%
Sensitivity Analysis 1	5,674	1,220.5	21.5%
Sensitivity Analysis 2	5,674	769.7	13.6%
Sensitivity Analysis 3	4,394	990.2	22.5%
Sensitivity Analysis 4	5,674	1112.2	19.6%
Sensitivity Analysis 5	4,394	893.6	20.3%

Table S5: Sample size and effective sample size of FIGARO-DKD by analysis

	**Sample size of the included FIGARO-DKD population**	**Effective sample size**	**Effective sample size /population**
Main Analysis	7352	1032.4	14.0%
Sensitivity Analysis 1	7352	988.5	13.4%
Sensitivity Analysis 2	7352	579.4	7.9%
Sensitivity Analysis 3	3537	171.0	4.8%
Sensitivity Analysis 4	7352	1008.8	13.7%
Sensitivity Analysis 5	3537	170.3	4.8%

Table S6: Finerenone and canagliflozin vs placebo: results of weighted analysis, additional endpoints

	**Finerenone vs placebo**			**Canagliflozin vs placebo**
**Endpoint**	**HR (95% CI) MAIC-weighted FIDELIO-DKD**	**HR (95% CI) MAIC-weighted FIGARO-DKD**	**HR (95% CI) M-A of FIDELIO-DKD and FIGARO-DKD**	**HR (95% CI), CREDENCE**
CV composite endpoint	0.82 (0.66; 1.03)	0.85 (0.63; 1.16)	0.83 (0.69; 0.99)	0.74 (0.63; 0.86)
Renal composite endpoint	0.73 (0.57; 0.92)	0.93 (0.58; 1.49)	0.77 (0.62; 0.95)	0.66 (0.53; 0.81)
Sustained eGFR decrease < 15 mL/min/1.73 m²	0.85 (0.66; 1.10)	0.96 (0.44; 2.11)	0.86 (0.67; 1.10)	0.60 (0.45; 0.80)
Non-fatal stroke	0.81 (0.51; 1.28)	0.86 (0.51; 1.43)	0.83 (0.59; 1.17)	0.80 (0.56; 1.15)
HF hospitalization	0.85 (0.60; 1.21)	0.82 (0.44; 1.53)	0.84 (0.62; 1.14)	0.61 (0.47; 0.80)
Non-fatal MI	0.87 (0.56; 1.34)	1.20 (0.62; 2.32)	0.96 (0.67; 1.38)	0.81 (0.59; 1.10)

CI, confidence interval; CV, cardiovascular; HR, hazard ratio; M-A, meta-analysis; MAIC, matching-adjusted indirect comparison

Endpoint definitions as in Supplementary Table 1

The HRs estimated for FIDELIO-DKD and FIGARO-DKD were based on a Cox proportional hazards model using the same covariates as in the respective finerenone study but applying a robust (“sandwich”) method of covariance estimation.

Table S7: Finerenone (FIDELIO-DKD + FIGARO-DKD) and canagliflozin results of MAIC, additional endpoints

Endpoint	MAIC: finerenone vs canagliflozin	
	HR (95% CI)	p-value
CV composite endpoint	1.12 (0.88; 1.42)	0.343
Renal composite endpoint	1.16 (0.86; 1.57)	0.327
Sustained eGFR decrease < 15 mL/min/1.73 m²	1.43 (0.98; 2.09)	0.061
Non-fatal stroke	1.04 (0.63; 1.71)	0.878
HF hospitalization	1.38 (0.92; 2.07)	0.118
Non-fatal MI	1.18 (0.73; 1.91)	0.489

CI, confidence interval; CV, cardiovascular; HR, hazard ratio; MAIC, matching-adjusted indirect comparison

Figure S1: Forest plot of MAIC finerenone vs canagliflozin for renal/cardiovascular composite endpoint

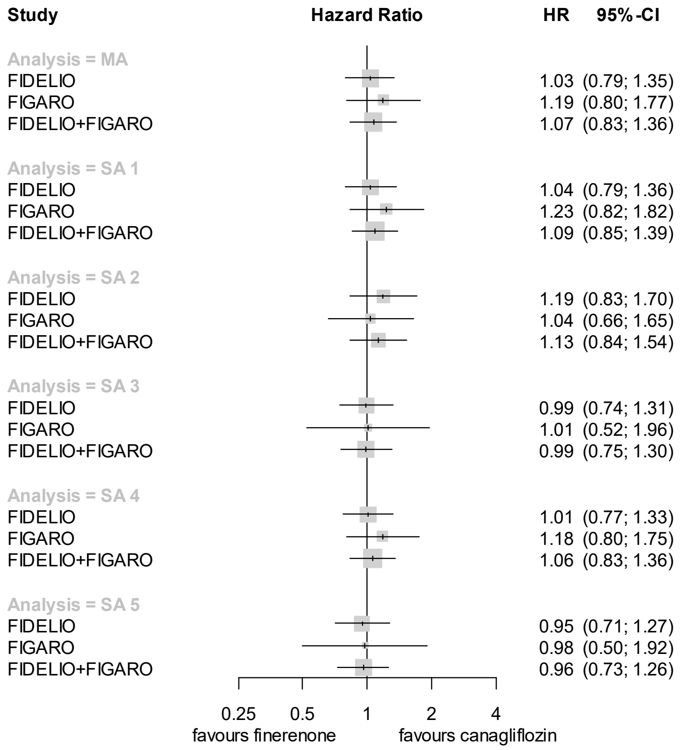

CI, confidence interval; HR, hazard ratio; SA, Sensitivity Analysis

Figure S2: Forest plot of MAIC finerenone vs canagliflozin for all-cause mortality endpoint

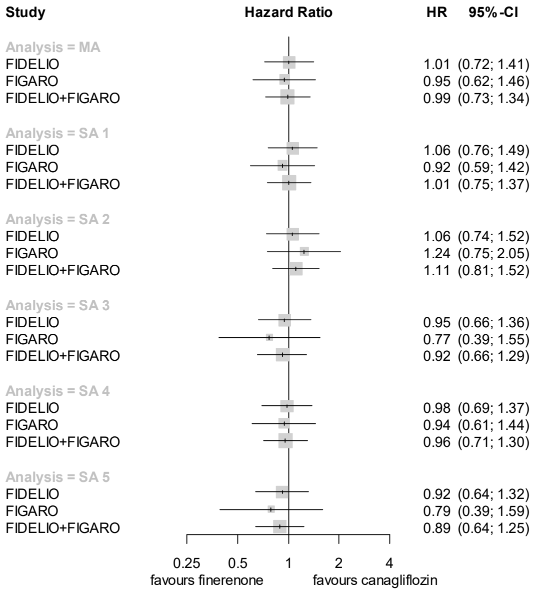

CI, confidence interval; HR, hazard ratio; MA, Main Analysis; SA, Sensitivity Analysis

Figure S3: Forest plot of MAIC finerenone vs canagliflozin for end-stage kidney disease endpoint

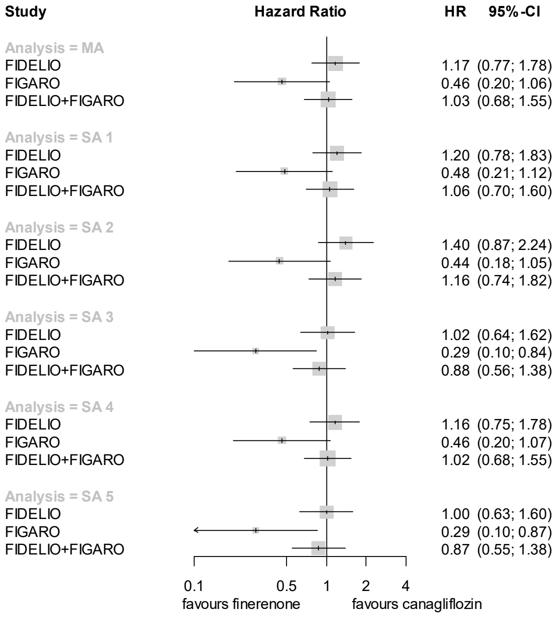

CI, confidence interval; HR, hazard ratio; MA, Main Analysis; SA, Sensitivity Analysis

Figure S4: Forest plot of MAIC finerenone vs canagliflozin for cardiovascular death endpoint

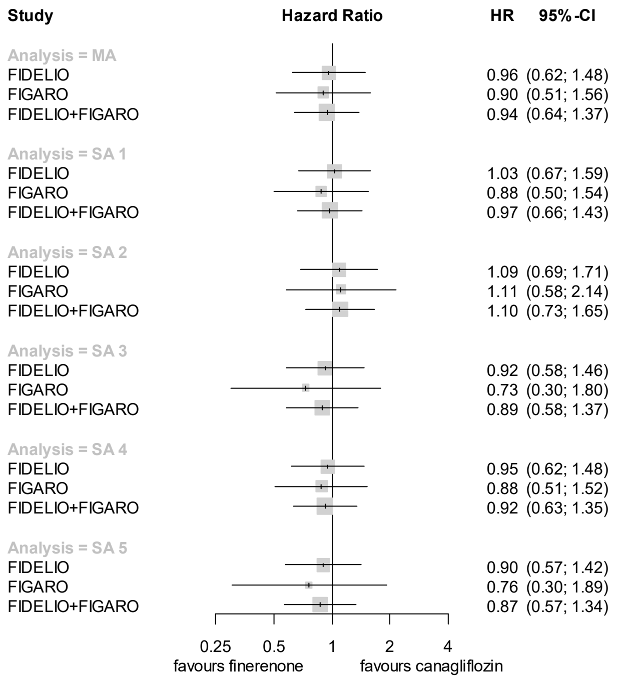

CI, confidence interval; HR, hazard ratio; MA, Main Analysis; SA, Sensitivity Analysis
